# The application of different appendicular skeletal muscle cutoff points and research definitions associated with health-related quality of life in Korean older people: data from KNHANES 2008–2011

**DOI:** 10.1186/1471-2318-14-144

**Published:** 2014-12-23

**Authors:** Yeon-Pyo Kim, Ju-Youn Joh, Sun Kim, Hwan-Sik Hwang, Il-Seon Shin

**Affiliations:** Department of Family Medicine, Chonnam National University Hwasun Hospital, 322, Seoyang-ro, Hwasun-eup, Hwasun-gun, Korea 519–809 Hwasun, Chonnam Korea; Center for Aging and Geriatrics, Chonnam National University Medical School, Gwang-Ju, Korea; Department of Psychiatry, Chonnam National University Hwasun Hospital, 322, Seoyang-ro, Hwasun-eup, Hwasun-gun, 519-809 Chonnam Korea

**Keywords:** Sarcopenia, Korean, Older people

## Abstract

**Background:**

This study was conducted to determine the prevalence of a low appendicular skeletal muscle index (ASMI) using three cut-off points (mean ASMI-2SD of a gender-specific young reference group (aged 18–39 years), mean ASMI-1SD of a gender-specific young reference group, and the lower 20 percentile value of a gender-specific older group (aged ≥ 65 years)) in Korean older people and the relationship between ASMI and subjective health-related quality of life.

**Methods:**

This study utilized data acquired during the Korean National Health and Nutrition Examination Survey (KNHANES) from 2008–2011. Dual-energy X-ray absorptiometry body compositional data was obtained from a subsample of 6538 subjects (men 2804, women 3734) aged 18–39 and 4413 subjects (men 1872, women 2541) aged 65 years and older. The three definitions of low appendicular skeletal muscle and the EQ-5D-3 L-Korean descriptive system were applied to Korean older people.

**Results:**

For the ASMI cutoff points used, in men, the three cutoff points were ASMI 2SD (6.09 kg/m^2^), ASMI 20 (6.48 kg/m^2^), and ASMI 1SD (6.95 kg/m^2^). In women, ASMI 2SD (4.38 kg/m^2^) was the lowest, followed by ASMI 1SD (4.96 kg/m^2^) and ASMI 20 (5.33 kg/m^2^). Proportions of older subjects with a low ASMI using the three cutoff points were 9.7% (ASMI 2SD) and 40.9% (ASMI 1SD) in men, and 0.7% (ASMI 2SD) and 7.4% (ASMI 1SD) in women. By multivariate ordinal logistic regression analysis, men with a low ASMI had significantly high odd ratios for the three domains of mobility (p < 0.001), self-care (p = 0.005), and usual activities (p = 0.004) among the five domains of the EQ-5D and EQ-5D index (p = 0.010).

**Conclusions:**

The ASMI 2SD cut-off points for older Koreans, 6.09 kg/m^2^ for men and 4.38 kg/m^2^ for women, resulted in low prevalences of a low ASM, that is, 9.7% for men and 0.7% for women, and showed low clinical usefulness due to very low determined prevalence in women. Hence, we suggest that the cut-off point of the lowest 20% of Korean older people (men: 6.48 kg/m^2^, women; 5.33 kg/m^2^) be used for older Koreans.

## Background

Life expectancies are increasing in most ethnic groups and countries, as are the survivals of older people with impaired physical functions and disabilities [1, 2]. Accordingly, there is a great deal of research interest in precursors, including sarcopenia, in older people with impaired physical functions and disabilities [3, 4].

Sarcopenia is a term applied by Irwin Rosenberg in 1989 to explain age-associated loss of muscle mass and muscle function [5]. Thereafter, researchers have added impaired functional performance and/or muscle weakness of clinical importance to the loss of muscle mass incorporated in the concept of sarcopenia. The Foundation for the National Institute of Health (FNIH) Sarcopenia Project defined sarcopenia as clinically relevant low muscle strength (weakness) and low lean mass in 2014 [6]. The Asian Working Group for Sarcopenia (AWGS) guideline also addressed cut-off points for Asian older people by modifying the European Working Group on Sarcopenia in Older People (EWGSOP) guideline [7].

The EWGSOP, International Working Group in Sarcopenia (IWGS) and AWGS guidelines define the sum of the muscle masses of the four limbs as appendicular skeletal mass (ASM) to calculate appendicular skeletal mass index (ASMI) as ASM/height^2^ (ASM/m^2^) [3, 4, 8–10]. However, many other cutoff points have been proposed [11]. Previous studies among Asian older people showed substantially different cutoff points for low ASMI. Furthermore, the prevalences of low ASMI among Asian women were extremely low, which caused problems for researchers conducting studies on sarcopenia.

This study was conducted to determine the prevalence of low ASMI in accordance with three cutoff points in aged Korean (>65 years), and to explore the relationship between low ASMI and subjective health-related quality of life.

## Methods

### Subjects

This study was based on data obtained from the 2008–2011 Korean National Health and Nutrition Examination Survey IV and V (KNHANES), a nationally-representative survey conducted by the Korean Ministry of Health and Welfare. KNHANES was a nationwide cross-sectional survey conducted in 1998–2012 by the Korean Center for Disease Control and Prevention (KCDC). KNHANES used stratified multi-stage clustered probability sampling to reflect the non-institutionalized Korean population. Participants completed a questionnaire that consisted of a health interview survey, a health behavior survey, a nutrition survey, and a health examination survey. A whole body dual energy X-ray absorptiometry (DXA) scan were performed on individuals ≥20 years old from July 2008 to June 2009 and on individuals of ≥10 years old from July 2009 to May 2011 (excluding pregnant women), individuals with a height of ≥196 cm or a weight of ≥136 kg were excluded in accord with the KNHANES survey protocol. In addition, test results were treated as missing value in participants with implanted radio-opaque material (e.g., a prosthetic device, implant or other radio-opaque object) that could affect DXA results [12].

After excluding those without a whole body DXA scan (aged 18–39 years: 123 men and 214 women, aged 65–97 years: 105 men and 143 women), 6538 subjects (aged 18–39 years: 2,804 men, 3,743 women; the young reference group) and 4413 subjects (aged 65–97 years: 1872 men, 2541 women; the main analysis group) were included in the final analysis. Written informed consent was given by all participants and the protocol for KNHANES IV and V were approved by the Institutional Review Board of the KCDC (2008-04EXP-01-C, 2009-01CON-03-2C, 2010-02CON-21-C, 2011-02CON-06-C). Current study did not require additional Institutional Review Board approval because the KNHANES data set is publicly available.

### DXA measurements

In the KNHANES study, whole body DXA examinations were conducted with a QDR4500A apparatus (Hologic, Bedford, MA, USA). All participants wore light clothing and removed all jewelry and other items that could interfere with DXA. The KNHANES data includes values for bone mineral content (g), bone mineral density (g/cm^2^), fat mass (g), lean mass (including bone mineral content (g)), and fat percent (%) for whole body and anatomical regions. From this data, ASM (kg) was calculated by summing the muscle masses of the four limbs, assuming that all non-fat and non-bone mass is skeletal muscle. ASMI was defined as ASM/height^2^ [13].

### Definition of low ASMI

The following exclusion criteria were applied to the 6538 eligible subjects (aged 18 to 39 years), a prior diagnoses or treatment for diabetes, stroke, angina, myocardial infarction, thyroid disease, arthritis, asthma, chronic obstructive pulmonary disease, depression, chronic renal disease, chronic viral hepatitis, liver cirrhosis, or any malignancy. In addition, subjects hospitalized for any reason during the past year were also excluded (n = 1835). A total of 4703 (men 2096, women 2607) were recruited for the young (18–39 years) reference group. Low ASMI groups were defined using the following three cutoff point points: ASMI 2SD - the cutoff value of the lowest 2.28% in gender-specific young reference groups, ASMI 1SD - the cutoff value of the lowest 15.87% in gender-specific young reference groups, and ASMI 20 - the cutoff value of the lowest 20% in gender-specific older study participants.

Unlike previous studies, ASMI values of the young reference group in the current study were not normally distributed. Because young women's data showed high kurtosis, we used approximate values (2.28%, and 15.87%) in place of 1SD and 2SD values, respectively. These approximate values can be used in non-normally distributed data, whereas values of 1SD and 2SD can be used in normally distributed data [14].

### EQ-5D-3 L -Korean (EQ-5D)

**Figure 1:**
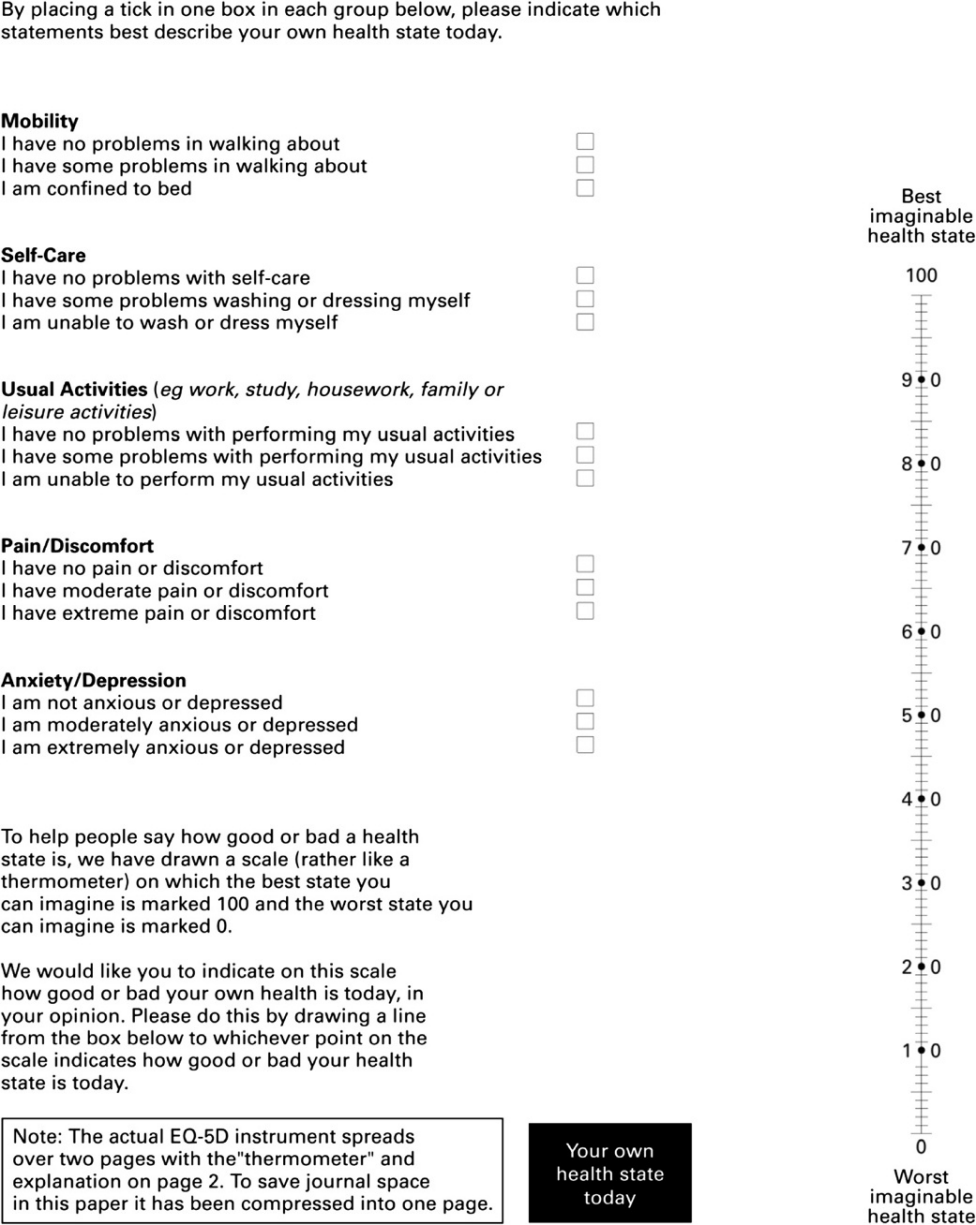
**EQ-5D 3 (EQ-5D) questionnaire.** Reproduced from [Journal of Neurology, Neurosurgery & Psychiatry, Schrag. A. et al., 69(1), 67–73, 2000] with permission from EuroQol Group and BMJ Publishing Group Ltd. [18].

To measure health-related quality of life, we used the European Quality of Life-5 Dimensions (EQ-5D) instrument of the EuroQol group [15]. Validity of Korean version of EQ-5D (Spearman correlation coefficient with the first question of the Health Survey Short-Form 36: -0.51 in EQ-5D and -0.52 in EQ-VAS) and reliability (test–retest reliabilities using intra-class correlation: 0.75 in EQ-5D and -0.77 in EQ-VAS; agreement reliability using kappa statistics between 0.46 and 0.77) have already been demonstrated [16]. EQ-5D measures five single-item dimensions (mobility, self-care, usual activities, pain/discomfort, and anxiety/depression), each item has three levels of response (no, some, or extreme problems) (Figure 1). Responses to the five items are used to derive an overall health index score (using the weighting). In this study, we used the weighting suggestion of Nam et al. [17].

### Covariates

The covariates included in the analysis were age, education, family income, body mass index (BMI), smoking status, alcohol consumption, chronic illness, and number of chronic illness. BMI was used to assess obesity as determined by the World Health Organization Asia-Pacific criteria: underweight (BMI <18.5 kg/m^2^), normal weight (BMI: 18.5-22.9 kg/m^2^), overweight (BMI: 23.0-27.4 kg/m^2^), and obese (BMI ≥27.5 kg/m^2^) [19]. Smoking status was categorized into four groups: never smokers (fewer than 100 cigarettes smoked during life), passive smokers (exposed to second-hand smoke for more than 1 hour a day in the home or workplace), current smokers, and former smokers. Alcohol consumption was assessed using the Korean version of the Alcohol Use Disorders Identification Test (AUDIT-K) and categorized based on scores into normal alcohol consumption group (men <9, women <5), harmful alcohol consumption group (men: 10–19, women: 6–9), and a suggestive alcohol dependent group (men >20, women >10) [20]. Chronic illnesses considered were cancer, diabetes, hypertension, stroke, coronary artery disease, arthritis, chronic pulmonary disease, depression, chronic renal failure, and chronic viral hepatitis/liver cirrhosis. These conditions were considered present when diagnosed by a physician at any time. We defined number of chronic illness as the sum of number of illness among the checklist of chronic illness.

### Statistical analyses

Statistical analyses were conducted using the complex sample procedure because the KNHANES data set was selected using a representative, stratified, and clustered sampling method, not a random sampling method. The data in Table 1 were analyzed after weighting, because we needed sample distributions, such as, SDs and percentile values, for detailed ASMI analysis. Using these data, we determined low ASMI group depending on ASMI 1SD and ASMI 2SD and ASMI 20 value. Thereafter, univariate and multivariate analyses and ordinal regression analysis was performed using the five domains of EQ-5D and EQ-5D index as dependent variables and ASMI groups as independent variables. Because of a previously reported gender effect between ASMI and clinical outcomes, univariate and multivariate analyses were performed in each gender group [6]. In multivariate analysis, factors associated with ADL or IADL in previous studies, status of obesity using BMI, smoking, AUDIT-K, chronic illness, and numbers of chronic disease, were considered additional independent variables. Education level and family income, which are known to be significantly associated with EQ-5D, were also considered independent variables [17]. Here a backward elimination procedure was used. Accordingly, to this procedure, nonsignificant covariates (p > 0.10) were removed from the model until a final solution was reached. Statistical significance was accepted for *p* values of <0.05. SPSS ver. 21.0 (SPSS, Chicago, IL, USA) was used for the statistical analysis.

**Table 1:** **Anthropometric characteristics of the healthy young reference group and older study group by gender**

	Men		Women	
	Young reference group	≥65 years	Young reference group	≥65 years
Sample size (no.)	2096	1872	2607	2541
Weighted size (no.)	6392302	2152083	5580819	3126111
Age (years)	29 (18–39)	71 (65–93)	29 (18–39)	72 (65–97)
Height (cm)	173.67 ± 5.651	164.81 ± 5.745	160.61 ± 5.406	150.47 ± 5.764
Weight (kg)	71.39 ± 11.241	62.81 ± 9.485	56.04 ± 9.520	54.62 ± 9.044
Waist circumference (cm)	81.29 ± 9.250	84.62 ± 9.036	72.44 ± 9.011	83.25 ± 9.562
Body mass index (kg/m^2^)	23.65 ± 3.415	23.07 ± 2.925	21.72 ± 3.495	24.07 ± 3.398
Total fat mass (kg)	15.54 ± 6.248	14.32 ± 4.896	17.81 ± 5.782	18.85 ± 5.651
Total body fat percentage (%)	21.40 ± 6.020	22.56 ± 5.434	31.47 ± 5.585	34.13 ± 5.935
ASM (kg)	23.60 ± 3.190	19.44 ± 2.756	14.66 ± 2.228	13.36 ± 1.905
ASMI (kg/m^2^)	7.81 ± 0.909	7.14 ± 0.811	5.67 ± 0.777	5.89 ± 0.663
ASMI 2SD cutoff point	6.09		4.38	
ASMI 1SD cutoff point	6.95		4.96	
ASMI 20 cutoff point		6.48		5.33

Weighted sample size reflects the number of objects and sample weights. Age is expressed as median (min-max). Other results are expressed as means ± SDs. ASM = appendicular skeletal muscle mass. ASMI = appendicular skeletal muscle index. Low ASMI groups were defined as subjects whose ASMIs were lower than the following three cut-off points: ASMI 2SD - the value of the lowest 2.28% of the gender-specific young reference group, ASMI 1SD - the value of the lowest 15.87% of the gender-specific young reference group, and ASMI 20 - the value of lowest 20% of the gender-specific old age study group.

## Results

Mean and SD of ASMI in the young men group assuming a normal distribution was 7.81 ± 0.91 kg/m^2^ and in the young women group was 5.67 ± 0.78 kg/m^2^. If ASMI in the young reference group had a normal distribution, the values of mean-2SD and the lowest 2.28% would have been the same. However, young men group (Kolmogorov-Smirnov p = 0.020) and the young women group (Kolmogorov-Smirnov p < 0.001) did not show a normal distribution due to high kurtosis. In the young men group, the mean-2SD value was 5.99 kg/m^2^ and the lowest 2.28% value was 6.09 kg/m^2^, and in the young women group corresponding values were 4.12 kg/m^2^ and 4.38 kg/m^2^, respectively. The difference between the two values was higher in the young women group (0.26 kg/m^2^ vs. 0.10 kg/m^2^). The ASMI cutoff points in older men were ASMI 2SD (6.09 kg/m^2^), ASMI 20 (6.48 kg/m^2^), and ASMI 1SD (6.95 kg/m^2^), and corresponding values for older women were ASMI 2SD (4.38 kg/m^2^), ASMI 1SD (4.96 kg/m^2^), and ASMI 20 (5.33 kg/m^2^) (Table 1). Characteristics of the older study participants are summarized in Table 2. The proportions in low group for each cutoff point for older men study subjects were 9.7% (ASMI 2SD) and 40.9% (ASMI 1SD), respectively, and for older women were 0.7% (ASMI 2SD) and 7.4% (ASMI 1SD), respectively (Table 3). By univariate analysis with ordinal regression analysis using the complex sample procedure, low ASMI had significantly associated with poorer outcome of five domains of EQ-5D and of the EQ-5D index in older men. More specifically, ASMI ranging from ASMI 2SD to ASMI 20 had higher cumulative odds ratios (ORs) than those with those a normal ASMI [mobility 2.28 (95% Confidence Interval (CI): 1.55-3.35), self-care (OR 2.10, CI: 1.10-4.00), usual activities (OR 2.40, CI: 1.53-3.77), pain/discomfort (OR 1.63, CI:1.13-2.36), anxiety/depression (OR 1.76, CI:1.01-3.09), and EQ-5D index (OR 2.09, CI: 1.45-3.02)] in older men (Table 4). Low ASMI had significantly associated with poorer outcome of domains of self-care and anxiety/depression among the five domains of EQ-5D in older women. More specifically, ASMIs ranging from ASMI 2SD to ASMI 20 had a higher OR for difficulty in self care (OR 1.20, CI: 0.82-1.74) and prevalence of anxiety/depression (OR 0.78, CI: 0.54-1.12) in older women (Table 5).

**Table 2:** **General characteristics of older study participants by gender**

		Men	Women
Sample size (no.)		1872	2541
Weighted size (no.)		2152083	3126111
Education			
	None	4.0 (0.6)	17.6 (1.2)
	Elementary school	24.1 (1.4)	45.3 (1.3)
	Middle school	29.1 (1.4)	26.7 (1.2)
	High school	20.3 (1.2)	6.6 (0.6)
	Over college grade	22.5 (1.4)	3.8 (0.5)
Family income			
	1st quartile	53.9 (1.2)	56.8 (1.4)
	2nd quartile	23.1 (0.8)	20.5 (1.0)
	3rd quartile	13.0 (0.8)	12.7 (0.9)
	4th quartile	9.9 (0.8)	10.0 (0.9)
BMI (kg/m^2^)			
	Underweight (<18.5)	6.1 (0.7)	3.9 (0.5)
	Normal weight (18.5-22.9)	43.5 (1.5)	33.8 (1.2)
	Overweight (23.0-27.4)	42.8 (1.4)	47.6 (1.3)
	Obese (>27.5)	7.5 (0.8)	14.7 (0.8)
Smoking			
	Never Smoking (<100 cigarettes in life)	17.9 (1.0)	85.8 (0.9)
	Passive smoking (>1 hour/day)	0.6 (0.2)	2.8 (0.4)
	Former smoker	27.8 (1.4)	2.4 (0.5)
	Current Smoker	53.7 (1.5)	9.0 (0.7)
Audit-K			
	Normal	70.5 (1.5)	92.8 (0.8)
	harmful alcohol consumption	22.4 (1.2)	4.6 (0.7)
	suggestive of alcohol dependence	7.1 (0.9)	2.6 (0.5)
Chronic illness			
	Cancer	6.4 (0.7)	5.8 (0.6)
	Diabetes	16.3 (0.9)	18.1 (1.0)
	Hypertension	41.4 (1.4)	54.4 (1.3)
	Stroke	7.3 (0.7)	4.2 (0.4)
	Coronary artery disease	6.3 (0.6)	5.2 (0.5)
	Arthritis	13.9 (1.0)	44.9 (1.2)
	Chronic Pulmonary Disease	6.0 (0.6)	6.7 (0.6)
	Depression	2.3 (0.4)	6.2 (0.6)
	Chronic renal failure	0.6 (0.2)	0.6 (0.1)
	Chronic hepatitis/Liver cirrhosis	2.5 (0.4)	1.2 (0.3)
No. of chronic illness		1.03 (0.98-1.09)	1.47 (1.42-1.53)

Weighted sample size reflects the number of objects and the sample weights. Results are expressed as percentages (standard errors). Numbers of chronic illnesses are expressed as means (95% Confidence Index).

**Table 3:** **Prevalence of low ASMI and EQ-5D for older study participants by gender**

			Men	Women
Sample size (no.)			1872	2541
Weighted size (no.)			2152083	3126111
ASMI				
	ASMI 2SD		9.7 (0.9)	0.7 (0.2)
	ASMI 1SD		40.9 (1.5)	7.4 (0.7)
	ASMI 20		20.0 (1.2)	20.0 (1.1)
EQ5D				
	Mobility			
		No problem	65.4 (1.4)	42.8 (1.3)
		Some problems	32.9 (1.3)	53.4 (1.3)
		Extreme problems	1.7 (0.4)	3.8 (0.5)
	Self-Care			
		No problem	90.4 (0.9)	80.9 (1.0)
		Some problems	8.6 (0.8)	17.4 (1.0)
		Extreme problems	1.0 (0.2)	1.7 (0.3)
	Usual Activities (e.g., work, study, housework, family or leisure activity)			
		No problem	76.6 (1.3)	61.9 (1.2)
		Some problems	19.9 (1.2)	31.5 (1.1)
		Extreme problems	3.5 (0.5)	6.6 (0.7)
	Pain/Discomfort			
		No problem	67.3 (1.3)	47.3 (1.3)
		Some problems	26.9 (1.2)	38.2 (1.2)
		Extreme problems	5.8 (0.6)	14.5 (0.8)
	Anxiety/Depression			
		No problem	88.9 (0.9)	79.5 (1.0)
		Some problems	9.3 (0.7)	17.4 (0.9)
		Extreme problems	1.9 (0.5)	3.2 (0.4)
	EQ5D index			
		1	52.6 (1.4)	28.9 (1.1)
		0.775-0.999	26.8 (1.3)	33.5 (1.3)
		<0.774	20.6 (1.2)	37.7 (1.3)

The weighted sample size reflects the number of objects and the sample weights. Results are expressed as percentages (standard errors). Low ASMI groups were defined as subjects whose ASMIs were lower than the following three cut-off points: ASMI 2SD - the value of the lowest 2.28% of the gender-specific young reference group, ASMI 1SD - the value of the lowest 15.87% of the gender-specific young reference group, and ASMI 20 - the value of lowest 20% of the gender-specific old age study group.

**Table 4:** **Univariate analysis of five domains of EQ-5D and EQ5D index according to ASMI in older men**

		Estimates	Standard error	Cumulative odds ratio	95% CI	p value
Mobility						<0.001*
	ASMI 2SD	0.818	0.192	2.267	1.554-3.308	<0.001
	ASMI 20	0.824	0.195	2.280	1.554-3.346	<0.001
	ASMI 1SD	0.311	0.159	1.365	0.998-1.867	0.051
	Normal	Reference				
Self-care						<0.001*
	ASMI 2SD	1.152	0.250	3.165	1.938-5.169	<0.001
	ASMI 20	0.743	0.328	2.103	1.104-4.007	0.024
	ASMI 1SD	0.252	0.294	1.286	0.721-2.294	0.393
	Normal	Reference				
Usual activities						<0.001*
	ASMI 2SD	1.153	0.195	3.169	2.159-4.651	<0.001
	ASMI 20	0.875	0.230	2.398	1.526-3.767	<0.001
	ASMI 1SD	0.500	0.181	1.648	1.156-2.350	<0.001
	Normal	Reference				
Pain/discomfort						0.010*
	ASMI 2SD	0.576	0.196	1.778	1.209-2.615	0.004
	ASMI 20	0.490	0.188	1.632	1.128-2.362	0.009
	ASMI 1SD	0.192	0.148	1.212	0.906-1.621	0.194
	Normal	Reference				
Anxiety/depression						0.012*
	ASMI 2SD	0.788	0.272	2.199	1.289-3.752	0.004
	ASMI 20	0.567	0.286	1.763	1.006-3.090	0.048
	ASMI 1SD	0.338	0.227	1.402	0.899-2.188	0.136
	Normal	Reference				
EQ5D index						<0.001*
	ASMI 2SD	0.879	0.161	2.408	1.755-3.304	<0.001
	ASMI 20	0.736	0.187	2.088	1.445-3.016	<0.001
	ASMI 1SD	0.305	0.142	1.357	1.028-1.792	0.031
	Normal	reference				

The weighted sample size reflects the number of objects and the sample weights. 95% CI: 95%.

Confidential Index for Cumulative Odds Ratio. *Adjustment for multiple comparisons was performed using the Sequential Sidak method. Low ASMI groups were defined as subjects whose ASMIs were lower than the following three cutoff points: ASMI 2SD - the value of the lowest 2.28% of the gender-specific young reference group, ASMI 1SD - the value of the lowest 15.87% of the gender-specific young reference group, and ASMI 20 - the value of lowest 20% of the gender-specific old age study group.

**Table 5:** **Univariate analysis of five domains of EQ-5D and EQ5D index according to ASMI in older women**

		Estimates	Standard error	Cumulative odds ratio	95% CI	p value
Mobility						0.264*
	ASMI 2SD	0.046	0.585	1.047	0.332-3.303	0.937
	ASMI 1SD	-0.102	0.227	0.903	0.579-1.410	0.654
	ASMI 20	-0.242	0.146	0.785	0.589-1.045	0.097
	Normal	Reference				
Self-care						0.011*
	ASMI 2SD	1.634	0.561	5.122	1.702-15.418	0.004
	ASMI 1SD	0.412	0.252	1.510	0.921-2.477	0.102
	ASMI 20	0.179	0.190	1.196	0.824-1.737	0.345
	Normal	Reference				
Usual activities						0.087*
	ASMI 2SD	1.238	0.569	3.450	1.129-10.542	0.030
	ASMI 1SD	0.188	0.220	1.206	0.784-1.857	0.394
	ASMI 20	0.231	0.157	1.260	0.924-1.716	0.143
	Normal	Reference				
Pain/discomfort						0.522*
	ASMI 2SD	1.101	0.894	3.009	0.520-17.416	0.218
	ASMI 1SD	0.057	0.191	1.058	0.728-1.539	0.767
	ASMI 20	-0.080	0.144	0.923	0.695-1.225	0.578
	Normal	Reference				
Anxiety/depression						0.021*
	ASMI 2SD	1.498	0.555	4.471	1.504-13.290	0.007
	ASMI 1SD	0.441	0.261	1.554	0.931-2.596	0.092
	ASMI 20	-0.253	0.188	0.776	0.536-1.123	0.179
	Normal	Reference				
EQ5D index						0.858*
	ASMI 2SD	0.637	0.898	1.890	0.324-11.034	0.479
	ASMI 1SD	0.087	0.211	1.091	0.721-1.650	0.681
	ASMI 20	-0.006	0.152	0.994	0.738-1.340	0.969
	Normal	Reference				

The weighted sample size reflects the number of objects and the sample weights. 95% CI: 95%.

Confidential Index for Cumulative Odds Ratio. *Adjustment for multiple comparisons was performed using the Sequential Sidak method. Low ASMI groups were defined as subjects whose ASMIs were lower than the following three cutoff points: ASMI 2SD - the value of the lowest 2.28% of the gender-specific young reference group, ASMI 1SD - the value of the lowest 15.87% of the gender-specific young reference group, and ASMI 20 - the value of lowest 20% of the gender-specific old age study group.

By multivariate ordinal logistic regression, low ASMI had significantly higher ORs for the three domains of mobility, self-care, and usual activities among the five domains of EQ-5D in older men (Table 4). In detail, the findings with ASMI 20 were mobility (OR 2.54, CI: 1.63-3.95), usual activities (OR 2.31, CI: 1.31-4.09), and EQ-5D index (OR 1.86, CI: 1.23-2.82) in older men (Table 6). However these correlations were not significant for all three cutoff points ASMI 2SD, ASMI 1SD (except ASMI 2SD), and ASMI 20 (except ASMI 1SD) in older women (Table 7).

**Table 6:** **Multivariate analysis of five domains of EQ-5D and EQ5D index according to ASMI in older men**

		Estimates	Standard error	Cumulative odds ratio	95% CI	p value
Mobility						<0.001*
	ASMI 2SD	0.698	0.272	2.010	1.117-3.431	0.011
	ASMI 20	0.932	0.225	2.538	1.632-3.948	<0.001
	ASMI 1SD	0.348	0.196	1.416	0.964-2.079	0.076
	Normal	Reference				
Self-care						0.005*
	ASMI 2SD	1.088	0.343	2.967	1.512-5.822	0.002
	ASMI 20	0.637	0.397	1.890	0.867-4.121	0.109
	ASMI 1SD	0.270	0.313	1.310	0.709-2.420	0.389
	Normal	Reference				
Usual activities						0.004*
	ASMI 2SD	1.082	0.335	2.950	1.526-5.701	0.001
	ASMI 20	0.839	0.290	2.313	1.309-4.087	0.004
	ASMI 1SD	0.539	0.213	1.714	1.128-2.606	0.012
	Normal	Reference				
Pain/discomfort						0.465*
	ASMI 2SD	0.385	0.292	1.469	0.828-2.609	0.188
	ASMI 20	0.241	0.243	1.272	0.790-2.049	0.322
	ASMI 1SD	0.113	0.180	1.120	0.786-1.595	0.530
	Normal	Reference				
Anxiety/depression						0.836*
	ASMI 2SD	0.140	0.352	1.150	0.576-2.295	0.691
	ASMI 20	0.277	0.374	1.319	0.633-2.750	0.460
	ASMI 1SD	0.199	0.264	1.220	0.726-2.050	0.453
	Normal	Reference				
EQ5D index						0.010*
	ASMI 2SD	0.656	0.247	1.928	1.187-3.132	0.008
	ASMI 20	0.622	0.212	1.863	1.229-2.824	0.003
	ASMI 1SD	0.204	0.168	1.227	0.882-1.707	0.224
	Normal	reference				

The weighted sample size reflects the number of objects and the sample weights. 95% CI: 95%.

Confidential Index for Cumulative Odds Ratio. *Adjustment for multiple comparisons was performed using the Sequential Sidak method for obesity criteria, family income, education, smoking, alcohol consumption criteria (AUDIT-K), chronic illness (cancer, diabetes, hypertension, stroke, coronary artery disease, arthritis, chronic pulmonary disease, depression, chronic renal failure, chronic viral hepatitis/ liver cirrhosis), and number of chronic diseases. Low ASMI groups were defined as subjects whose ASMIs were lower than the following three cutoff points: ASMI 2SD - the value of the lowest 2.28% of the gender-specific young reference group, ASMI 1SD - the value of the lowest 15.87% of the gender-specific young reference group, and ASMI 20 - the value of lowest 20% of the gender-specific old age study group.

**Table 7:** **Multivariate analysis of five domains of EQ-5D and EQ5D index according to ASMI in older women**

		Estimates	Standard error	Cumulative odds ratio	95% CI	p value
Mobility						0.093*
	ASMI 2SD	-1.407	0.826	0.245	0.048-1.240	0.089
	ASMI 1SD	0.157	0.299	1.170	0.650-2.106	0.599
	ASMI 20	-0.469	0.218	0.626	0.408-0.961	0.032
	Normal					
Self-care						0.974*
	ASMI 2SD	-0.227	1.265	0.797	0.066-9.561	0.858
	ASMI 1SD	0.136	0.360	1.146	0.565-2.325	0.705
	ASMI 20	-0.042	0.288	0.959	0.544-1.689	0.884
	Normal					
Usual activities						0.710*
	ASMI 2SD	-0.111	1.354	0.895	0.063-12.791	0.935
	ASMI 1SD	0.315	0.328	1.370	0.719-2.610	0.338
	ASMI 20	-0.014	0.228	0.986	0.630-1.544	0.951
	Normal					
Pain/discomfort						0.299*
	ASMI 2SD	-1.274	1.375	0.280	0.019-4.165	0.354
	ASMI 1SD	-0.082	0.287	0.921	0.524-1.619	0.775
	ASMI 20	-0.306	0.192	0.737	0.505-1.074	0.112
	Normal					
Anxiety/depression						0.754*
	ASMI 2SD	-0.754	1.210	0.470	0.044-5.070	0.534
	ASMI 1SD	0.177	0.309	1.194	0.651-2.190	0.566
	ASMI 20	-0.248	0.279	0.780	0.451-1.349	0.374
	Normal					
EQ5D index						0.490*
	ASMI 2SD	-1.828	1.427	0.161	0.010-2.652	0.201
	ASMI 1SD	0.113	0.317	1.120	0.601-2.085	0.721
	ASMI 20	-0.182	0.234	0.833	0.527-1.319	0.436
	Normal					

The weighted sample size reflects the number of objects and the sample weights. 95% CI: 95%.

Confidential Index for Cumulative Odds Ratio. *Adjustment for multiple comparisons was performed using the Sequential Sidak method for obesity criteria, family income, education, smoking, alcohol consumption criteria (AUDIT-K), chronic illness (cancer, diabetes, hypertension, stroke, coronary artery disease, arthritis, chronic pulmonary disease, depression, chronic renal failure, chronic viral hepatitis/ liver cirrhosis), and Number of chronic disease. Low ASMI groups were defined as subjects whose ASMIs were lower than the following three cutoff points: ASMI 2SD - the value of the lowest 2.28% of the gender-specific young reference group, ASMI 1SD - the value of the lowest 15.87% of the gender-specific young reference group, and ASMI 20 - the value of lowest 20% of the gender-specific old age study group.

## Discussion

In this Korean national representative sample, the prevalences of low ASMI according to a cutoff point of ASMI 2SD were 9.7% and 0.7% for older (≥65 years) men and women, respectively, however, according to cutoff point of ASMI 1SD, 40.9% and 7.4% of the older men and women, respectively, had a low ASMI. In addition, only older men with a low ASMI were found to have a low health-related quality of life.

The means and SDs of ASMI were 7.14 ± 0.81 kg/m^2^ and 5.89 ± 0.66 kg/m^2^ in older Korean men and women, respectively, which are a little lower than those of previous studies. The New Mexico Elder Health Survey for Hispanic and non-Hispanic Americans conducted from May 1993 to September 1995 reported a mean ASMI was 7.7 ± 0.7 kg/m^2^ in older men and 5.9 ± 0.7 kg/m^2^ in older women [13]. The Health ABC study on Americans conducted from 1997 to 1998 reported a mean ASMI in European Americans of 7.8 ± 0.9 kg/m^2^ in older men and of 6.1 ± 0.8 kg/m^2^ in older women [21]. A Japanese study conducted from July 2002 to June 2003 reported mean ASMIs of 7.61 ± 0.7 kg/m^2^ in older men and 6.32 ± 0.6 kg/m^2^ in older women [22]. Finally, a study in Taiwan published in 2013 reported mean ASMIs of 7.6 ± 0.8 kg/m^2^ and 6.4 ± 0.6 kg/m^2^ in older men and women, respectively [23].

On the other hand, mean ASMIs in the young reference group in the present study were 7.81 ± 0.91 kg/m^2^ and 5.67 ± 0.78 kg/m^2^ in men and women, which are markedly lower than the values obtained in the American study, which reported mean ASMIs for a young reference group (18–40 years) of 8.6 ± 1.1 kg/m^2^ and 7.3 ± 0.9 kg/m^2^ in men and women [13]. The present findings are also lower than those in the Taiwanese study, which reported mean ASMIs in a disease-free young reference group (20–40 years) of 8.2 ± 0.9 kg/m^2^ and 5.9 ± 0.8 kg/m^2^ in men and women [23].

In The New Mexico Elder Health Survey for Hispanic and non-Hispanic Americans, the mean ASMIs of older men and women were approximately at the 87 and 80 percentiles, respectively, of the young reference group [13]. In our study, the mean ASMIs of older men and women were at the 91 and 104 percentiles, of the young reference group, and the mean ASMI of older women was higher than the mean ASMI of the young woman reference group.

The proportion of older people with an ASMI two standard deviations below the mean ASMI of the young reference group also displayed a marked difference in the present study as compared with previously published values. In our study, older people with below ASMI 2SD were 9.7% in older men and 0.7% in older women, as compared to 13% at 61 to 70 years of age, 24% at 71 to 80 years of age, and 50% at aged 81 aged and older [13], and 28% in men ≥70 years of age and 52% in women ≥70 years of age [24].

Interestingly, many studies on sarcopenia in Asian populations have not used the standard ASMI cutoff of two standard deviations below the mean ASMI of the young reference group. The ASMI 1SD cutoff points used in Japanese and Korean studies were 6.53 kg/m^2^ in men and 5.21 kg/m^2^ in women and 7.09 kg/m^2^ in men and 5.27 kg/m^2^ in women, while a study in Taiwan used the lower 20 percentile values of gender specific healthy young age individuals [22, 23, 25]. Considering the diagnostic criteria of sarcopenia recommended by the IWGS, EWGSOP, and AWGS, these sets of research standards are atypical. It is possible that the use of the ASMI 2SD value as a cutoff point in the aforementioned studies would have resulted in an unacceptably small proportion of sarcopenic older individuals. Other studies in Asians may have been similarly hampered.

A number of reasons can be suggested for the low proportions of older people with an ASMI 2SD below the mean ASMI of young reference groups in the present study. First, the body types of Koreans are changing due to improvements in living standards. According to the 2010 Size-Korea survey of body measurement, the average height of Koreans increased steadily up to 2003 and remained constant thereafter, whereas the body types of Koreans have consistently changed. In that survey, the proportion of body shape of Korean 20s women changed to 7.3-head units tall as compared with 20 years earlier a 7.2-head units tall, and leg lengths of Korean women were nearly 2 centimeters longer than 30 years earlier [26]. Second, Korean women in their 20s and 30s can have poor weight reducing habits, and rely diet restriction rather than exercise. This reflects a distorted body image, with a desire to have a body type similar to Caucasian women, Korean women tend to prefer a slim body with focus on diet restriction, have a negative effect on muscle development [27–29]. As a result, muscle mass in Korean women has been consistently reported to be decreasing more so than body fat because of repetitive low calorie diets and accompanying cycles of weight loss and weight gain [27, 30, 31]. These results are consistent with the results of the Size-Korea survey, which found that mean BMI of subjects in their late 20s significantly decreased over time and the proportion of underweight individuals (as determined by BMI) increased to 15.7% in 2010 from 11.5% in 2003, while decreases in waist circumference was not significant over the same period [26]. As a result, in the present study, the mean ASMI of the young reference women group was lower than the mean ASMI of the older women group (≥65 years of age) and the proportion of older women in the below ASMI 2SD group was excessively low.

Because ASMI values of the young reference group in the current study did not show a normal distribution, we used alternative values that corresponded to lowest 2.28% and 15.87% values in the young reference group in place of 1SD and 2SD values, respectively. After making this adjustment, the cutoff point of ASMI 2SD increased from 4.12 kg/m^2^ to 4.38 kg/m^2^ in older women, and had we applied the real ASMI 2SD value (4.12 kg/m^2^) only four older women would have had a low ASMI.

It is suggested that the existing sarcopenia diagnostic criteria using ASMI of a young reference group of the same gender and race is inadequate for Koreans. Considering clinical outcomes later in life, re-establishment of optimal ASMI cut-off points is needed.

We performed multivariate ordinal logistic regression analysis to examine the association between the low ASMI group based on the three established ASMI cutoff points mentioned and EQ-5D determined health-related quality of life. Low ASMI had significantly associated with poorer outcome of three domains of mobility, self-care, and usual activities among the five domains of the EQ-5D in older men. However, in older women with a low ASMI, these correlations were not significant for any of the three ASMI cutoff points. These findings are consistent with previous finding of a stronger correlation between sarcopenia and functional decline and disability in older men [4]. Additional studies about gender-associated differences in muscle aging are needed.

Although the association between the low ASMI group based on ASMI 20 and any domain of EQ-5D was not significant in older women, the ASMI 20 cutoff point was significantly associated with EQ-5D in older men. On the other hand, the ASMI 2SD and ASMI 1SD cutoff points may not be adequate to diagnose sarcopenia in older Koreans because the proportions of older people with sarcopenia are quite variable. Studies are needed on the optimal ASMI cutoff point with respect to muscle function and clinical outcome for Koreans. We suggest that an ASMI 20 cutoff point (men 6.48 kg/m^2^, women 5.33 kg/m^2^) is appropriate for older people for the assessment of low ASM, until an optimal cutoff point is determined. Further studies on country/population specific ASMI cutoff point are needed in different communities.

This study had several strengths. First, our data were collected from a nationwide survey that reflected the community-dwelling Korean population. In particular, because the young reference group was large (2096 men and 2607 women) and was nationally representative, the ASMI distribution based on the young reference group can be considered reliable. Second, we performed analyses in a gender-specific manner. We found that the association between height-adjusted ASM and EQ-5D differed in older men and women, which is consistent with the findings of the recent FNIH study, in which height-adjusted ASM was found to be more useful for men and weight-adjusted ASM for women with respect skeletal muscle functions [6], implying a possible underlying gender effect.

Finally, to our best knowledge, this study is the first report of a significant association between low ASMI and health-related quality of life. Our finding implies a possible relationship between sarcopenia and health-related quality of life, however, further studies are required to confirm the relation.

On the other hand, the present study has following limitations that warrant consideration. First, the study lacked investigation of cause-effect relationships because of its cross-sectional nature. Future prospective cohort studies are needed for such investigations. Second, previous studies analyzed ADL and IADL as dependent variables, whereas we used EQ-5D as a dependent variable. Although EQ-5D contains items similar to ADL and IADL, such as, mobility, self-care, and usual activities, direct comparisons might be inappropriate. Third, we did not consider muscle strength and muscle function and we used only height-adjusted ASM. The recent guidelines (EWGSOP, IWGS, AWGS and FNIH guideline) recommend to diagnose sarcopenia as reduced muscle mass related to muscle strength and function such as grip strength and gait speed, not only reduced muscle mass itself. However, our KNHANES data set did not contain information on muscle strength and muscle function, thus we have analysed only muscle mass in this study. Further studies are needed using our suggested ASM cutoff point and the FNIH guidelines, which represent the state of the art, recommend BMI-adjusted ASM. Finally, we did not include physical activity as a potential confounder because KNHANES IV and KNHANES V had different items of measurement. Further studies on the topic are needed.

## Conclusions

Among Korean older people, the prevalence of low ASM was 9.7% in men and only 0.7% in women when an ASMI 2SD cutoff point of 6.09 kg/m^2^ was used for men and 4.38 kg/m^2^ for women, which lowers its clinical usefulness because of the extremely low determined prevalence in women. Hence, we suggest that the cutoff point of the lowest 20% of Korean older people (men: 6.48 kg/m^2^, women; 5.33 kg/m^2^) be used for older Korean people.
